# MicroRNA-643 regulates the expression of ZEB1 and inhibits tumorigenesis in osteosarcoma

**DOI:** 10.3892/mmr.2022.12792

**Published:** 2022-07-11

**Authors:** Huan Wang, Danmou Xing, Dong Ren, Wei Feng, Yan Chen, Zhiming Zhao, Zhihong Xiao, Zhengren Peng

Mol Med Rep 16: 5157–5164, 2017; DOI: 10.3892/mmr.2017.7273

Following the publication of this paper, it was drawn to the Editors’ attention by a concerned reader that certain of the cell invasion and migration assay data shown in Fig. 2E and 4E, and the immunohistochemical data shown in Fig. 5C were strikingly similar to data appearing in different form in other articles by different authors. Owing to the fact that the contentious data in the above article had already been published elsewhere, or were under consideration for publication, prior to its submission to *Molecular Medicine Reports*, the Editor has decided that this paper should be retracted from the Journal. After having been in contact with the authors, they agreed with the decision to retract the paper. The Editor apologizes to the readership for any inconvenience caused.

